# A Thoroughly Validated Virtual Screening Strategy for Discovery of Novel HDAC3 Inhibitors

**DOI:** 10.3390/ijms18010137

**Published:** 2017-01-18

**Authors:** Huabin Hu, Jie Xia, Dongmei Wang, Xiang Simon Wang, Song Wu

**Affiliations:** 1 State Key Laboratory of Bioactive Substance and Function of Natural Medicines, Department of New Drug Research and Development, Institute of Materia Medica, Chinese Academy of Medical Sciences and Peking Union Medical College, Beijing 100050, China; dreaming@imm.ac.cn (H.H.); jiexia@imm.ac.cn (J.X.); wangdmchina@imm.ac.cn (D.W.); 2 Molecular Modeling and Drug Discovery Core for District of Columbia Center for AIDS Research (DC CFAR), Department of Pharmaceutical Sciences, College of Pharmacy, Howard University, Washington, DC 20059, USA; x.simon.wang@gmail.com

**Keywords:** HDAC3 inhibitors, anti-diabetes, virtual screening, MUBD-HDACs, benchmarking calculation

## Abstract

Histone deacetylase 3 (HDAC3) has been recently identified as a potential target for the treatment of cancer and other diseases, such as chronic inflammation, neurodegenerative diseases, and diabetes. Virtual screening (VS) is currently a routine technique for hit identification, but its success depends on rational development of VS strategies. To facilitate this process, we applied our previously released benchmarking dataset, i.e., MUBD-HDAC3 to the evaluation of structure-based VS (SBVS) and ligand-based VS (LBVS) combinatorial approaches. We have identified FRED (Chemgauss4) docking against a structural model of HDAC3, i.e., SAHA-3 generated by a computationally inexpensive “flexible docking”, as the best SBVS approach and a common feature pharmacophore model, i.e., Hypo1 generated by Catalyst/HipHop as the optimal model for LBVS. We then developed a pipeline that was composed of Hypo1, FRED (Chemgauss4), and SAHA-3 sequentially, and demonstrated that it was superior to other combinations in terms of ligand enrichment. In summary, we present the first highly-validated, rationally-designed VS strategy specific to HDAC3 inhibitor discovery. The constructed pipeline is publicly accessible for the scientific community to identify novel HDAC3 inhibitors in a time-efficient and cost-effective way.

## 1. Introduction

Histone deacetylases (HDACs) are a family of enzymes that catalyze histone deacetylation, a major form of posttranslational modifications in mammals. HDACs work together with their counterpart, i.e., histone acetyltransfereases (HATs), to maintain acetylation homeostasis and regulate gene transcription [1,2,3,4]. Functional abnormality of HDACs or/and HATs could cause dysregulation of associated genes and, thus, result in a wide range of diseases, including cancers, neurodegenerative diseases (e.g., Alzheimer’s disease) and other types of diseases (e.g., inflammatory diseases) [5,6]. Mammalian HDACs include 18 isoforms, and they are classified into four classes according to sequence homology to yeast HDACs, i.e., Class I (HDAC1, HDAC2, HDAC3, HDAC8), class II (IIA: HDAC4, HDAC5, HDAC7 and HDAC9; IIB: HDAC6 and HDAC10), class III (i.e., Sirtuins, SIRT1-SIRT7), and class IV (HDAC11).

In the whole family of HDACs, HDAC3 has gained more and more attention in recent years and showing its potential as a target for modern drug discovery. HDAC3 maintains the primarily deacetylated state of nuclear factor κB (NF-κB), which is a positive regulator of inflammatory gene expression [7]. Due to this, HDAC3 plays an important role in inflammatory responses. Moreover, the inhibition of HDAC3 brings about anti-inflammatory effects and is, thus, beneficial for the treatment of many chronic inflammatory diseases, such as airway inflammation and inflammatory bowel diseases [6,8].

**Figure 1:**
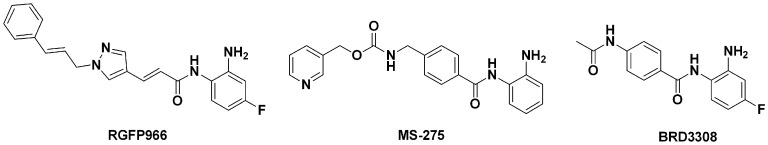
The chemical structures of RGFP966, MS-275, and BRD3308.

In recent years, the relationship between HDAC3 and learning/memory has been gradually uncovered. Wood’s group identified HDAC3 as a negative regulator of long-term memory formation, and demonstrated HDAC3 inhibition was able to improve cognitive impairments [9]. They also discovered HDAC3 negatively regulated cocaine-context-associated memory formation [10], and the HDAC3-selective inhibitor RGFP966 (cf. Figure 1) was able to persistently enhance extinction of cocaine-seeking behavior [11]. Therefore, HDAC3 inhibitors (HDAC3Is) are potentially applicable to the treatment of neurodegenerative diseases and the control of drug addiction.

In addition, growing evidence suggests a link between HDACs and the pathophysiology of diabetes. All HDACs, except for class III (SIRT1–SIRT7), are expressed in the pancreatic β-cells [12]. The inhibition of HDACs potentially prevents diabetes or reverts it to the normal state through a variety of mechanisms. For instance, it promotes β-cell development, proliferation, differentiation, and also prevents β-cell apoptosis [13,14,15,16,17]. In addition, it improves oxidative metabolism, ameliorates the state of insulin resistance, as well as regulates liver gluconeogenesis [18]. However, cytotoxicity has been observed, as well, in the use of HDAC inhibitors, e.g., SAHA [19,20]. Up to now, great efforts have been undertaken to discriminate the individual HDAC(s) for anti-diabetic benefits from others that cause side effects. Most importantly, Wagner’s group demonstrated it was sufficient to suppress β-cell apoptosis by genetic knockdown of *Hdac3* alone or by using the HDAC1/2/3-selective inhibitor, MS-275 (cf. Figure 1) [19]. They further developed a set of pharmacological tools that had different inhibitory profiles for HDACs, e.g., HDAC3-selective inhibitor, BRD3308 (cf. Figure 1) and HDAC1/2-selective inhibitor, BRD2492. Comparative analysis of their effects on β-cell survival and megakaryocyte formation (a surrogate measure of bone marrow toxicity) has identified HDAC3 instead of HDAC1/2 as a potential therapeutic target for β-cell protection [20]. Encouragingly, BRD3308 has also been shown to improve glycaemia and insulin secretion in vivo [21].

Currently, many ligands are able to inhibit HDAC3. However, most of them belong to anti-cancer pan-HDAC inhibitors and rarely show specific inhibition for HDAC3. To the best of our knowledge, RGFP966 and BRD3308 are the only HDAC3-specific inhibitors that show therapeutic effects on diseases other than cancers. Therefore, it remains an emerging area to discover novel HDAC3Is for the treatment of those diseases. We have been working on identification of novel-scaffold HDAC inhibitors by using computer-aided drug design (CADD) and cheminformatics [22,23]. Most recently, we developed an automated tool, i.e., MUBD-DecoyMaker for building benchmarking sets able to unbiasedly evaluate ligand enrichment of both ligand-based VS (LBVS) and structure-based VS (SBVS) approaches [24,25]. With that tool, we constructed maximal-unbiased benchmarking datasets (MUBD) for HDACs (including Sirtuins), i.e., MUBD-HDACs and released them in order to facilitate HDAC inhibitors discovery [5]. Up to now, the application of MUBD-HDACs has effectively assisted Huang et al., to identify a novel and potent HDAC inhibitor that showed anti-cancer activity [26]. In the extant paper, we present a versatile pipeline that is able to effectively enrich HDAC3-targeted active compounds from large-scale chemical libraries. To develop that pipeline, we use one dataset of MUBD-HDACs, i.e., MUBD-HDAC3, to exhaustively evaluate a variety of SBVS and LBVS approaches, including docking programs, scoring functions, and ligand-induced-fit protein models, as well as multiple pharmacophore/shape-based models. The constructed pipeline will be helpful for the scientific community to identify novel HDAC3Is in a time-efficient and cost-effective way.

## 2. Results and Discussions

### 2.1. Structure-Based VS (SBVS) Approaches

#### 2.1.1. The Optimal Docking Program and Scoring Function

Table 1 shows ligand enrichments of three docking programs, i.e., LibDock, GOLD, and FRED. GOLD (Chemscore) was the weakest docking program in terms of both early recognition and overall enrichment. Its values of receiver operating characteristic (ROC) enrichment at 0.5% (i.e., ROCE 0.5%), ROCE 1%, and ROC AUC (area under the curve) were 0, 0, and 0.63, respectively. LibDock (LibScore) ranked in second place. Though its value of ROC AUC was slightly higher than that of FRED (Chemgauss4), its ROCE 0.5% and ROCE 1% values were much lower, i.e., 10.89 vs. 30.90 and 5.42 vs. 25.66. Based on this outcome, FRED (Chemgauss4) was the optimal docking program to enrich for active ligands.

We explored the potentials of 10 scoring functions implemented in DS 2016 (Discovery Studio version 2016, San Diego, CA, USA) to improve ligand enrichment. As FRED performed the best among all programs, all docking poses from it became the input for the “score ligand poses” module in DS. Unfortunately, this rescoring approach did not further improve ligand enrichment of FRED (Chemgauss4), as all ROCE 0.5%, ROCE 1%, and ROC AUC values of those explored scoring functions were lower than those of FRED (Chemgauss4) (cf. Table 1).

#### 2.1.2. The Optimal Protein Models from Ligand-Induced-Fit Docking

**Figure 2:**
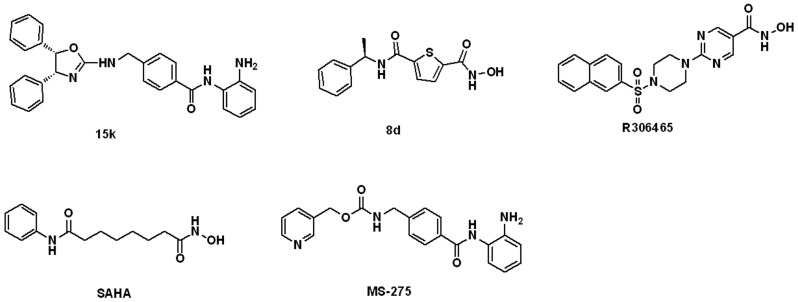
The chemical structures of five HDAC3Is in the ligand set for molecular modeling.

In addition to docking programs and scoring functions, we hypothesized that ligand-induced-fit protein models may enrich HDAC3Is better that the apo structure (i.e., a structure without a ligand). To test this hypothesis, we generated models by ligand-induced-fit docking of five HDAC3Is (cf. Figure 2) into the catalytic site. As a result, flexible docking generated five protein-ligand complexes for 15k, 26 for 8d, 34 for R306465, 40 for SAHA, and 32 for MS-275. By visual inspection, we excluded the complexes that apparently did not contain a coordination bond between zinc ion and the aminoanilide or hydroxamic acid groups. The numbers of protein-ligand complexes for 15k, 8d, R306465, SAHA, and MS-275 were reduced as a result to 5, 12, 8, 12, and 5, respectively.

**Figure 3:**
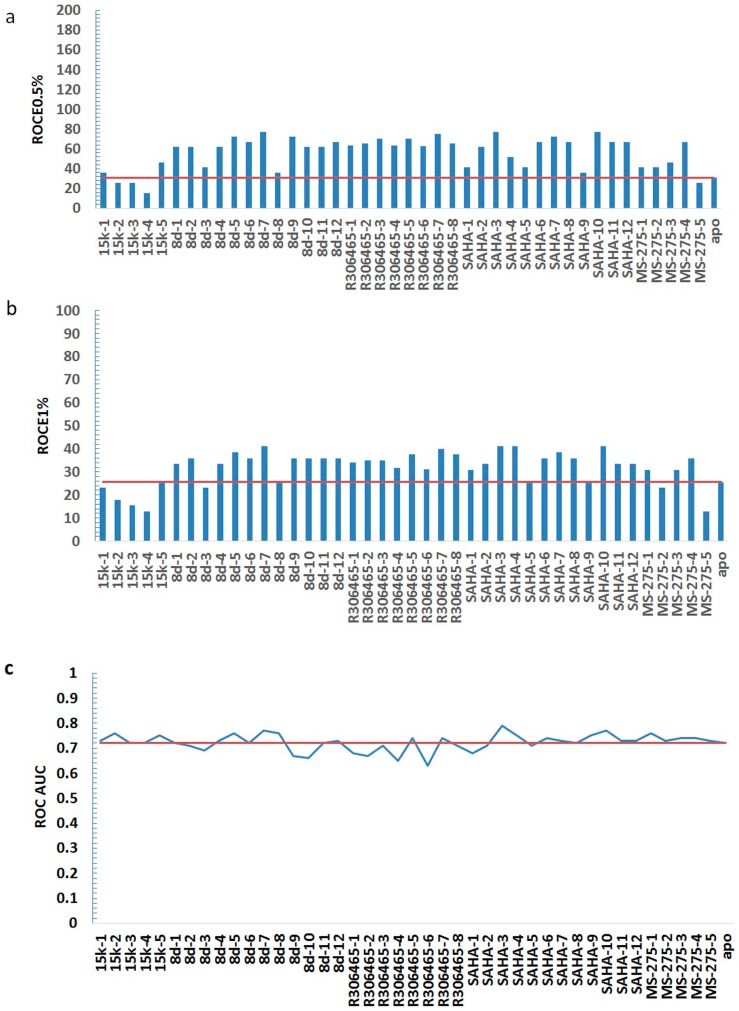
Ligand enrichments of 42 ligand-induced-fit models as well as the crystal structure of HDAC3 in the metrics of ROCE 0.5% (**a**); ROCE 1% (**b**); and ROC AUC (**c**). The red line represents the value of the apo crystal structure (i.e., structure without a ligand) of HDAC3.

The total number of ligand-induced-fit protein models was 42. As FRED (Chemgauss4) was validated as the best docking/scoring program, it was used to dock and score all actives and decoys in MUBD-HDAC3 against every ligand-induced-fit model. Ligand enrichments of these models along with the use of FRED (Chemgauss4) are shown in Figure 3 and Table 2. Generally, ROCE 0.5% and ROCE 1% values of most protein models were higher than the apo structure, while ROC AUC values remained equivalent to that of the apo structure (cf. Figure 3). To be specific, the average ROCE 0.5% and ROCE 1% values of all models were 56.55 and 31.83, both of which were greater than those values of the apo crystal structure, i.e., 30.90 and 25.66. Meanwhile, the average of ROC AUC was equal to that of apo structure, i.e., 0.72 (cf. Table 2). Overall, these data indicate the induced-fit effect from flexible docking was able to improve early recognition and maintained overall enrichment of the apo structure.

**Figure 4:**
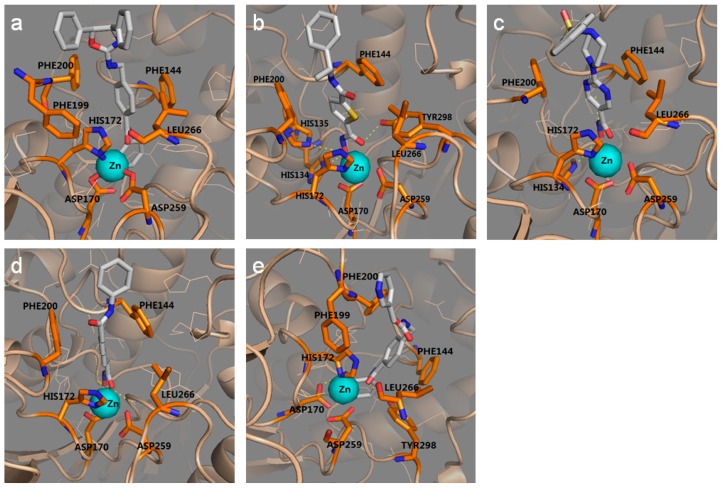
The interaction between five HDAC3Is and the optimal protein models obtained from ligand-induced-fit docking. (**a**) 15k/15k-5; (**b**) 8d/8d-7; (**c**) R306465/R306465-7; (**d**) SAHA/SAHA-3; and (**e**) MS-275/MS-275-4. Color codes: orange, residues involved in protein–ligand interaction; light blue, zinc ion; gray, ligand.

We further analyzed the ligand-induced-fit protein models from the same ligand and selected the best model, i.e., model of highest enrichment for each ligand. As shown in Table 2, the best models induced by those ligands are 15k-5, 8d-7, R306465-7, SAHA-3, and MS-275-4. Among them, both 8d-7 and SAHA-3 presented the highest early enrichment (77.25 for ROCE 0.5% and 41.05 for ROCE 1%), but SAHA-3 showed slightly better performance in term of overall enrichment (0.79 vs. 0.77). Apart from ligand enrichment, we also inspected the binding mode between each ligand and its induced-fit protein structure (cf. Figure 4a–e). Though differences existed in their binding modes, they shared common features: (1) a zinc ion was located in a cavity composed of His172, Asp170, and Asp259, and it interacted with the aminoanilide or hydroxamic acid group of each ligand; (2) the hydrophobic group in the middle of each ligand was placed between Phe200 and Phe144. Such observations prompted us to generate common feature pharmacophore models for LBVS.

The above data confirmed that inclusion of ligand-induced-fit effect by “flexible docking” in DS was able to generate protein-ligand complexes that (1) contained essential interactions with a zinc ion; and (2) more importantly, possessed better ligand enrichment than the apo structure. In addition, the use of a benchmarking calculation based on MUBD-HDAC3 was effective in selecting the optimal structural model.

### 2.2. Ligand-Based VS (LBVS) Approaches

#### 2.2.1. Pharmacophore Models Generated by Catalyst/HipHop

Since five HDAC3Is in the ligand set for molecular modeling were observed to share common features in different protein-ligand complexes, we used Catalyst/HipHop to generate common feature pharmacophore models. According to Table 3, (1) all of the models were composed of four pharmacophore features, though there were two classes, i.e., RHDA and RHAA; (2) the rank scores of all models were equivalent, in a range from 42.530 to 46.740; (3) Hypo1 performed the best according to rank score, but not all ligands in the ligand set fully matched Hypo1 as its direct hit was 11101. By contrast, the scores of Hypo4 and Hypo9 ranked at the fourth and ninth places, but all ligands were able to fully match these two models. Therefore, we were unable to determine an optimal model from Hypo1and Hypo4/Hypo9 based on these preliminary data.

**Figure 5:**
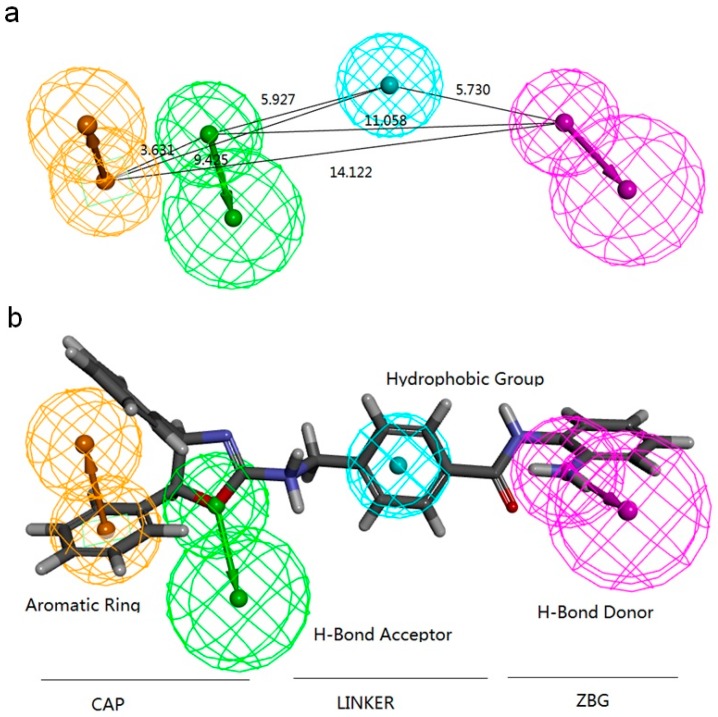
The optimal pharmacophore model for the HDAC3 inhibitor, i.e., Hypo1 generated by Catalyst/HipHop. (**a**) Distance (Å) between each feature of Hypo1; and (**b**) a representative compound in the ligand set, i.e., 15k mapped to Hypo1. Color codes: orange, aromatic ring; green, hydrogen bond acceptor; light blue, hydrophobic group; magenta, hydrogen bond donor.

Ligand enrichment is a preferred metric for predicting model performance in real-world screening. The evaluation outcome based on MUBD-HDAC3 is listed in Table 4. Hypo1 was superior to all other models, including Hypo4 and Hypo9, to which all five ligands were able to fully match. Based on both ligand enrichment and rank score, Hypo1 is undoubtedly the best among all models generated by Catalyst/HipHop. Figure 5 illustrates the details of Hypo1, including four pharmacophore features and their pairwise distances. As an example, the most potent HDAC3I in the ligand set, i.e., 15k, was mapped to Hypo1. According to both the pharmacophore model and the above predicted binding mode (cf. Figure 4a), compounds active for HDAC3 should contain (1) a group that includes a hydrogen bond donor on one side; (2) a hydrophobic group in the middle; (3) a group that contains an aromatic ring and a hydrogen-bond acceptor on the other side. As defined by others, these regions were the zinc binding group (ZBG) for (1); linker for (2); and a capping group for (3) [27,28].

Interestingly, we observed the inconsistency between rank and enrichment (cf. Table 3 and Table 4) for a few models. For instance, ligand enrichment (ROCE1%) of Hypo2 was not as good as that of Hypo3, though its rank score is better. The same situation happened with Hypo4 and Hypo5. Such inconsistency indicates the better rank score from Catalyst/HipHop does not necessarily represent better enrichment, which highlights the necessity of a benchmarking calculation.

#### 2.2.2. Shape-Based Models Generated by ROCS

**Figure 6:**
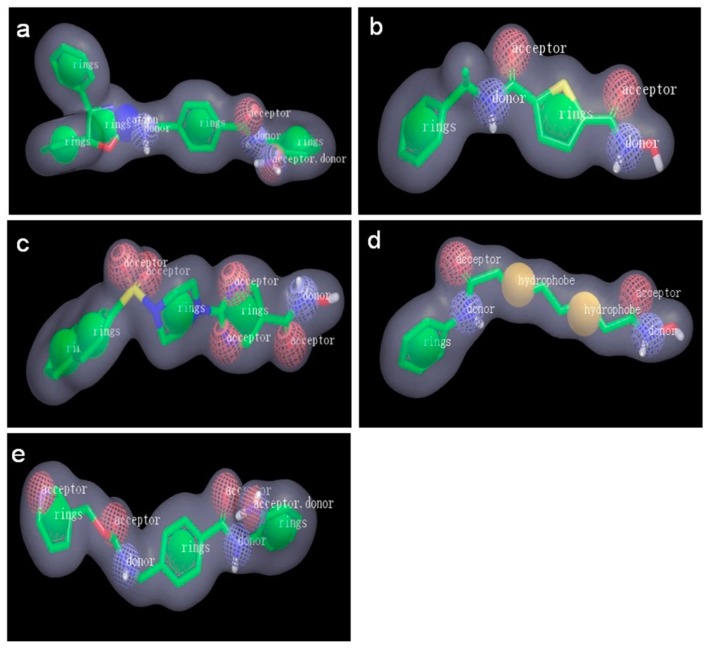
Five shape-based models/queries generated by ROCS. (**a**) 15k; (**b**) 8d; (**c**) R306465; (**d**) SAHA; and (**e**) MS-275. Color codes: yellow, hydrophobic group; green, aromatic ring; blue, hydrogen bond donor; red, hydrogen bond acceptor; gray, shape feature.

In addition to common features, five HDAC3Is in the ligand set for molecular modeling had their own features in terms of both chemical structure and binding pose. ROCS program was applied to generate models to uncover these differences. Figure 6 shows shape-based models/queries (including pharmacophore features) based on five poses extracted from protein-ligand complexes, i.e., 15k-5L, 8d-7L, R306465-7L, SAHA-3L, and MS-275-4L. Notably, five models displayed unique shapes as well as unique pharmacophore features. We used these models to screen MUBD-HDAC3 and applied five different similarity metrics in ROCS, i.e., TanimotoCombo (TC), ShapeTanimoto (ST), ColorTanimoto (CT), ScaledColor (SC), and ComboScore (CS), to score actives/decoys in MUBD-HDAC3. The resultant ligand enrichment from each similarity metric is listed in Table 5.

As for ROC AUC, the values for all pairs of model/metric are all around 0.50 and their pairwise difference is within 0.10. This indicated in general that ligand enrichment of a shape-based similarity search was no better than in random enrichment. However, all pairs of models/metrics, except for SAHA-3L/ST (ROCE 0.5%: 0; ROCE 1%: 0), performed better in early recognition than random selection. The best metric varies for each model. The best metric for 15k-5L was TC, a combined score of ST and CT. With the use of CT, 8d-7L showed best enrichment. The metrics that result in optimal enrichments for R306465-7L, SAHA-3L and MS-275-4L are CT, TC, and CS (combined score of ST and SC), respectively. Among them, the best pair was SAHA-3L/TC, whose early enrichments in terms of ROCE 0.5% and ROCE 1% were 20.60 and 17.96. Nevertheless, it was still not as good as Hypo1 from Catalyst/HipHop (ROCE 0.5%: 41.20; ROCE 1%: 23.09; ROC AUC: 0.86).

### 2.3. The Optimal Strategy to Virtually Screen HDAC3Is

Via exhaustive evaluation, we have identified (1) the ligand-induced-fit protein model, i.e., SAHA-3 combined with FRED (Chemgauss4) led to best ligand enrichment, in particular early recognition; (2) the common feature pharmacophore model, i.e., Hypo1 generated by Catalyst/HipHop was superior to shape-based models generated by ROCS; and (3) The optimal SBVS approach performed better than pharmacophore filtering, i.e., Hypo1. Based on the outcome, we developed a pipeline to rapidly and effectively screen large-scale chemical libraries. This pipeline was composed of pharmacophore filtering by Hypo1, followed by use of FRED docking against SAHA-3. Notably, this combination showed better performance than the use of Hypo1 alone. Meanwhile, it did not significantly impair the good early recognition of molecular docking alone, as its ROCE 0.5% and ROCE 1% values remained close, i.e., 72.10 and 38.49 (cf. Table 6). In light of both computational cost and screening power, we deemed Hypo1_FRED_SAHA-3 was the optimal strategy to virtually screen for HDAC3Is.

**Figure 7:**
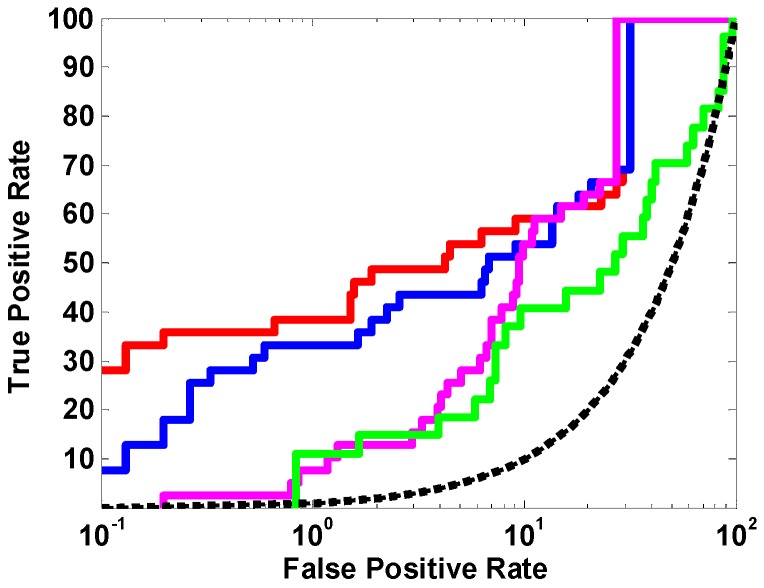
The ROC curves of four pipelines. Color codes: red, Hypo1_FRED_SAHA-3; blue, hypo1_FRED_Apo; magenta, Hypo1_LibDock_Apo; green, Hypo1_GOLD_Apo; black, random.

In addition, we evaluated three other combined pipelines, i.e., Hypo1_GOLD_apo, Hypo1_LibDock_apo, and Hypo1_FRED_apo. These pipelines were the most probably constructed and applied ones for HDAC3Is discovery, if ligand enrichment assessment (i.e., benchmarking calculation) was not performed. The reasons were: (1) Hypo1 had a high rank score in Catalyst/HipHop and, thus, would undoubtedly become the first component of the discovery pipelines (cf. Table 3); (2) any of the commonly used docking programs, i.e., GOLD, LibDock, and FRED, would be arbitrarily selected due to the lack of feasible metrics; and (3) the only available apo structure for HDAC3 would be used as the receptor for docking. According to Table 6, the three pipelines were all inferior to Hypo1_FRED_SAHA-3 in terms of ligand enrichment. The ROC curves clearly illustrate the same outcome (cf. Figure 7). These data validated the advantages of our methods, e.g., flexible docking and benchmarking calculation, in the construction of an optimal pipeline.

## 3. Materials and Methods

### 3.1. Data Collection and Curation

#### 3.1.1. The Ligand Set for Molecular Modeling

Five potent and diverse HDAC3Is were selected from the literature, i.e., 15k [29], 8d [30], R306465 [31], SAHA [32], and MS-275 [32] (cf. Figure 2). This set of HDAC3Is covered two major types of scaffolds, i.e., aminoanilide and hydroxamic acid. Prior to molecular modeling, these ligands were protonated at the pH range of 7.3–7.5 by using the “Prepare Ligands” module in DS 2016 (Dassault Systèmes BIOVIA, San Diego, CA, USA). In the present study, this ligand set was utilized to induce multiple protein models by flexible docking. Also, it was used to generate common feature pharmacophore models by Catalyst/HipHop.

#### 3.1.2. The Benchmarking Set for Ligand Enrichment Assessment

As mentioned before, MUBD-HDACs can be applied to benchmark both LBVS and SBVS approaches in an unbiased manner [5]. Therefore, the dataset designed specific to HDAC3, i.e., MUBD-HDAC3 was used to evaluate ligand enrichment of all approaches in this study, including docking programs, scoring functions, ligand-induced-fit protein models, and pharmacophore/shape-based models. MUBD-HDAC3 was downloaded from Wang’s lab (available on: http://www.xswlab.org/, accessed in January 2016) [33]. It included 39 diverse HDAC3Is, 1521 unbiased decoys, as well as a ready-to-use apo structure for HDAC3 (PDB Entry: 4A69).

### 3.2. Metrics to Assess Ligand Enrichment

Based on a list of ranked scores of ligands and decoys in MUBD-HDAC3, a receiver operating characteristic (ROC) curve was generated to evaluate ligand enrichment of different approaches. A ROC curve is a plot that describes the true positive rate against the false positive rate at various discrimination threshold settings. It is commonly used in data analysis to evaluate the performance of a binary classifier in discriminating the positive (active) from the negative (decoy). The more specific metrics from the ROC curve were ROC area under the curve (ROC AUC) and ROC enrichment (ROCE) values. The former represented overall enrichment, while the latter measured early recognition. Since early recognition has more practical significance than overall enrichment in real-world screening campaigns, ROCE 1% and ROCE 0.5% were given priority for determining the optimal approach. [34] The ROCE value was defined as the quotient of true positive rate divided by the false positive rate at a given percentage of binding decoys, i.e., 0.5% or 1%. In the present study, those actives or decoys that failed in pose generation or molecular docking were relisted with a maximum/minimum score. This ensured a fair comparison of different approaches in the benchmarking calculation.

### 3.3. Development and Evaluation of SBVS Approaches

#### 3.3.1. Three Commonly Used Docking Programs

Three docking programs that we had access to, LibDock implemented in DS, GOLD (version 3.0.1, Cambridge Crystallographic Data Center, Cambridge, UK), and FRED (now OEDocking, version 3.0.1, OpenEye Scientific Software, Inc., Santa Fe, NM, USA) were applied to screen MUBD-HDAC3. Each program had its inherent scoring function, i.e., Libscore for LibDock, Chemscore for GOLD, and Chemgauss4 for FRED. The ready-to-use apo structure from MUBD-HDAC3 was directly used as a receptor for molecular docking. In molecular docking simulations using the three programs, zinc ion in the binding site was treated as rigid.

For LibDock, the binding site was defined as a sphere centered on zinc ion in the catalytic site with a radius of 12 Å. All compounds in MUBD-HDAC3 were converted to 3D conformers by the “FAST” method in DS, with 255 as the maximum conformations and 20 kcal/mol as an energy threshold. All conformers were docked against the binding site and scored by Libscore. A maximum of 30 poses were saved for each compound. For GOLD, it was not necessary to generate a multi-conformer database before molecular docking. This program used the initial low-energy conformation of each compound as an input, and applied a genetic algorithm to optimize the pose in the binding site. The binding site was defined as exactly the same as that for LibDock. The zinc ion was retained and predefined to form tetrahedral coordination geometries during molecular docking. The docking mode was set as “7–8 times speed-up” and each compound was docked 30 times. All poses were scored by ChemScore. For molecular docking by FRED, firstly OMEGA [35] (version 2.5.1.4, OpenEye Scientific Software, Inc.) was utilized to build a multi-conformer database for all the compounds in MUBD-HDAC3 with default parameters. Then, as GOLD was able to deal with coordination with the zinc ion, a representative HDAC3I, such as 15k, was “positioned” in the catalytic site by GOLD and its best scoring pose was used to define the binding site in a form of grid box. Subsequently, FRED docked all compounds from the multi-conformer database into the binding site. After that, Chemgauss4 scored all docking poses and 30 top scoring poses of each compound were kept.

For the benchmarking calculation, only the best docking score of every ligand/decoy was selected. Based on the list of scores, ROC AUC and ROCE 0.5%/1% values were calculated for each docking program.

#### 3.3.2. Other Structure-Based Scoring Functions

As different scoring functions show inconsistent “screening power” [36,37], rescoring using other functions may have the potential to further improve ligand enrichment of the optimal docking program. Herein, 10 scoring functions available in DS were explored: LigScore1, LigScore2, PLP1, PLP2, Jain, PMF, PMF04, LUDI1, LUDI2, and LUDI3. Based on the evaluation outcome of three docking programs, all poses from the optimal docking program were submitted to the “Score Ligand Poses” module of DS. Again, the binding site was defined as a sphere centered on the zinc ion in the catalytic site with a radius of 12 Å. For each scoring function, the top scoring pose of each compound was retained for the benchmarking calculation.

#### 3.3.3. Multiple Ligand-Induced-Fit Protein Models

Receptor flexibility is also a factor that may affect ligand enrichment. Therefore, the apo structure of HDAC3 was an “induced fit” with every HDAC3I in the ligand set (cf. Figure 2). Prior to the induced fit, GOLD was used to generate a best scoring pose that coordinated with zinc ion. With that as the starting pose, the ligand was docked against the apo structure of HDAC3 by using “flexible docking” module in DS [38]. In flexible docking, the binding site was defined as a sphere with a radius of 12 Å centered on the zinc ion. Those amino acid residues covered by the binding site were set as removable, which became the basis for the generation of receptor conformations and refinement of side chains. Other parameters were set as default. After flexible docking, the generated protein-ligand complexes were visually inspected. Those complexes that apparently did not contain interactions between the aminoanilide or hydroxamic acid group of the ligand and zinc ion were excluded. In the remaining complexes, all ligand poses were stripped and the corresponding receptor confirmations, i.e., ligand-induced-fit protein models were retained. Then, the optimal docking program and scoring function selected from the last step were applied to dock MUBD-HDAC3 against these models. Based on docking scores of actives and decoys in MUBD-HDAC3, the ligand enrichment of each model was calculated.

### 3.4. Development and Evaluation of LBVS Approaches

#### 3.4.1. Pharmacophore Modeling: Catalyst/HipHop

The Catalyst/HipHop module implemented in DS was applied to generate common feature pharmacophore models based on the ligand set (cf. Figure 2). Prior to pharmacophore modeling, two attributes of the ligands, i.e., Principal and MaxOmitFeat, were defined. The former attribute indicates whether the ligand is active (2), moderately active (1), or inactive (0), while the latter represents the maximum number of omitted features. For 15k and 8d, Principal and MaxOmitFeat were set to 2 and 0, respectively. For the other three HDAC3Is, both Principal and MaxOmitFeat were set to 1. For each ligand, the FAST method was used to quickly generate a maximum of 255 diverse low-energy conformations, with 20 kcal/mol as an energy threshold. Hydrogen-bond acceptor (A), hydrogen-bond donor (D), hydrophobic group (H), and ring aromatic (R) were predefined as potential features in pharmacophore models. The minimum and maximum counts of each feature were set to 0 and 5. Based on the above parameters, 10 pharmacophore models were automatically generated. Then, the resultant pharmacophore models were scored (ranked) by Catalyst/HipHop. The rank is a measure of how well the ligands map onto the pharmacophore model.

As ligand enrichment is the most significant metric for VS, these models were further evaluated. Retrospective small-scale VS using each pharmacophore model was performed based on the benchmarking set, i.e., MUBD-HDAC3. Actives and decoys in MUBD-HDAC3 were converted to 3D conformers by using the DS module “Build 3D Database” and then screened by using the DS module of “Search 3D Database”. Based on fit values of all compounds, ROC curves were generated and corresponding parameters, i.e., ROCE and ROC AUC, from the curves were obtained.

#### 3.4.2. Shape-Based Models: ROCS

ROCS (version 3.0.1, OpenEye Scientific Software Inc., Santa Fe, NM, USA) is a shape-based superposition method and well known for its accuracy and speed [39,40]. Based on every ligand pose whose complementary protein model possesses best enrichment of all models induced by that ligand, ROCS generated five shape-based models (including pharmacophore features) using the default Implicit-Mills force field. No additional editing was applied to these models. These models were then evaluated for ligand enrichment by the benchmarking calculation, i.e., retrospective small-scale VS based on MUBD-HDAC3. To be specific, all of the actives and decoys in MUBD-HDAC3 were converted to a multi-conformer database by OMEGA and screened/scored by five different similarity metrics that included TanimotoCombo (TC), ShapeTanimoto (ST), ColorTanimoto (CT), ScaledColor (SC), and ComboScore (CS). For each metric, the best scoring pose was retained for each compound. Based on the scores for each metric, ROC curves and parameters were calculated.

### 3.5. Development of Pipeline for Large-Scale VS

Due to low computational cost, LBVS approaches are usually set up ahead of SBVS approaches in a pipeline for large-scale VS. Accordingly, the best model obtained from the evaluation of LBVS approaches and the optimal SBVS approaches, e.g., docking program/scoring function and the ligand-induce-fit protein model, were integrated into a pipeline. This pipeline was then benchmarked based on the MUBD-HDAC3 dataset. To do this, the compounds in MUBD-HDAC3 were filtered by the best pharmacophore/shape-based model. Those compounds that passed the filter were further docked against the optimal structural model of HDAC3 by the selected docking program and scored by the best scoring function. For the purpose of comparison, those compounds that passed the filter were also docked against the apo structure using a common docking/scoring program. The outcomes from these combined LBVS and SBVS approaches were then analyzed.

## 4. Conclusions

In this paper, we used MUBD-HDAC3 to unbiasedly validate the screening power of various SBVS and LBVS approaches and select the optimal ones. Stepwise evaluation of docking programs and scoring functions identified FRED (Chemgauss4) as optimal for enriching HDAC3Is based on the apo structure. Docking against multiple ligand-induced-fit protein models by FRED (Chemgauss4) further uncovered SAHA-3 as a structural model of better screening power. Comparative analysis of common feature pharmacophore models by Catalyst/HipHop and shape-based models by ROCS demonstrated Hypo1 from the former performed the best in terms of early enrichment. Based on the outcome, we presented a versatile pipeline, i.e., Hypo1_FRED_SAHA-3, to rapidly and effectively enrich HDAC3-targeted active compounds from large-scale chemical libraries. We have demonstrated this rationally developed pipeline was superior to other combinations in terms of ligand enrichment, and this highlighted the importance of benchmarking calculations in decision-making.

To the best of our knowledge, this work is the first to attempt to develop VS pipelines for intentionally screening HDAC3-specific inhibitors. The models associated with this VS pipeline, i.e., Hypo1 and SAHA-3, and their usages are publicly accessible via the following link (available on: https://www.dropbox.com/sh/5w9d6y8m77hk9pb/AAA2QoItV7wEeFS4p1ZyQzMda?dl=0). Our immediate goal is to apply this pipeline to screen chemical libraries from different sources and test potential hits for their HDAC potency/selectivity profile as well as their anti-diabetic effect.

## Figures and Tables

**Table 1:** Ligand enrichments of three docking programs and 10 scoring functions.

LBVS Approach		ROCE 0.5% ^a^	ROCE 1% ^a^	ROC AUC ^b^
docking programs	GOLD (Chemscore)	0.00	0.00	0.63
	LibDock (LibScore)	10.89	5.42	0.77
	FRED (Chemgauss4)	30.90	25.66	0.72
other scoring functions	Ludi_1 (DS ^c^)	0.00	0.00	0.39
	Ludi_2 (DS)	0.00	0.00	0.40
	Ludi_3 (DS)	0.00	0.00	0.33
	Ligscore1 (DS)	0.00	2.57	0.56
	Ligscore2 (DS)	0.00	0.00	0.37
	PLP1 (DS)	5.15	7.70	0.50
	PLP2 (DS)	10.30	5.13	0.53
	Jain (DS)	5.15	2.57	0.40
	PMF (DS)	5.15	5.13	0.54
	PMF04 (DS)	0.00	5.13	0.52

^a^ The quotient of the true positive rate divided by the false positive rate at top-ranked 0.5% (for ROCE 0.5%) or 1% (for ROCE 1%) of binding decoys. A greater value indicates a better early recognition of a docking/scoring approach; ^b^ area under the ROC curve. A greater value represents a better overall enrichment; ^c^ Discovery Studio.

**Table 2:** Ligand enrichments of 42 ligand-induced-fit models as well as the crystal structure of HDAC3.

HDAC3I	Model	ROCE 0.5%	ROCE 1%	ROC AUC
No ligand	apo	30.90	25.66	0.72
15k	15k-1	36.05	23.09	0.73
	15k-2	25.75	17.96	0.76
	15k-3	25.75	15.40	0.72
	15k-4	15.45	12.83	0.72
	15k-5	46.35	25.66	0.75
8d	8d-1	61.80	33.36	0.72
	8d-2	61.80	35.92	0.71
	8d-3	41.20	23.09	0.69
	8d-4	61.80	33.36	0.73
	8d-5	72.10	38.49	0.76
	8d-6	66.95	35.92	0.72
	8d-7	77.25	41.05	0.77
	8d-8	36.05	25.66	0.76
	8d-9	72.10	35.92	0.67
	8d-10	61.80	35.92	0.66
	8d-11	61.80	35.92	0.72
	8d-12	66.95	35.92	0.73
R306465	R306465-1	63.69	34.17	0.68
	R306465-2	65.28	35.02	0.67
	R306465-3	70.30	35.02	0.71
	R306465-4	63.69	31.73	0.65
	R306465-5	70.30	37.53	0.74
	R306465-6	62.49	31.13	0.63
	R306465-7	75.32	40.03	0.74
	R306465-8	65.28	37.53	0.71
SAHA	SAHA-1	41.20	30.79	0.68
	SAHA-2	61.80	33.36	0.71
	SAHA-3	77.25	41.05	0.79
	SAHA-4	51.50	41.05	0.75
	SAHA-5	41.20	25.66	0.71
	SAHA-6	66.95	35.92	0.74
	SAHA-7	72.10	38.49	0.73
	SAHA-8	66.95	35.92	0.72
	SAHA-9	36.05	25.66	0.75
	SAHA-10	77.25	41.05	0.77
	SAHA-11	66.95	33.36	0.73
	SAHA-12	66.95	33.36	0.73
MS-275	MS-275-1	41.20	30.79	0.76
	MS-275-2	41.20	23.09	0.73
	MS-275-3	46.35	30.79	0.74
	MS-275-4	66.95	35.92	0.74
	MS-275-5	25.75	12.83	0.73
statistics	average	56.55	31.83	0.72
	min	15.45	12.83	0.63
	max	77.25	41.05	0.79

**Table 3:** Ten pharmacophore models generated by Catalyst/HipHop and their ranks.

Model	Features ^a^	Rank	Direct Hit ^b^	Partial Hit ^c^	Max Fit
Hypo1	RHDA	46.740	11101	00010	4
Hypo2	RHDA	46.725	11101	00010	4
Hypo3	RHDA	46.725	11101	00010	4
Hypo4	RHDA	44.288	11111	00000	4
Hypo5	RHDA	44.206	11101	00010	4
Hypo6	RHDA	44.206	11101	00010	4
Hypo7	RHDA	44.039	11101	00010	4
Hypo8	RHDA	44.039	11101	00010	4
Hypo9	RHAA	43.379	11111	00000	4
Hypo10	RHAA	42.530	11101	00010	4

^a^ R = Aromatic Ring; H = Hydrophobic group; A = H-Bond Acceptor; D = H-Bond Donor; ^b^ Direct Hit: the ligands that fully match the pharmacophore. 11101, all ligands except for the fourth one, directly match the pharmacophore; ^c^ Partial Hit: the ligands that partially match the pharmacophore. 00010, the fourth ligand partially matched the pharmacophore.

**Table 4:** Ligand Enrichments of 10 pharmacophore models in terms of ROCE 0.5%, ROCE 1%, and ROC AUC.

Model	ROCE 0.5%	ROCE 1%	ROC AUC
Hypo1	41.20	23.09	0.86
Hypo2	20.60	12.83	0.85
Hypo3	20.60	17.96	0.85
Hypo4	0.00	0.00	0.59
Hypo5	10.30	7.70	0.76
Hypo6	10.30	12.83	0.77
Hypo7	5.15	5.13	0.74
Hypo8	0.00	5.13	0.73
Hypo9	5.15	2.57	0.55
Hypo10	5.15	2.57	0.69

**Table 5:** Ligand enrichments of five shape-based models in terms of ROCE 0.5%, ROCE 1%, and ROC AUC.

Model	Similarity Metric *	ROCE 0.5%	ROCE 1%	ROC AUC
15k-5L	TC	15.45	10.26	0.55
	ST	10.30	7.70	0.50
	CT	5.15	7.70	0.59
	SC	10.30	7.70	0.53
	CS	15.45	7.70	0.52
8d-7L	TC	20.60	10.26	0.44
	ST	15.45	7.70	0.43
	CT	20.60	12.83	0.48
	SC	15.45	7.70	0.47
	CS	15.45	7.70	0.43
R306465-7L	TC	10.30	7.70	0.54
	ST	5.15	2.57	0.5
	CT	10.30	10.26	0.55
	SC	15.45	7.70	0.52
	CS	10.30	7.70	0.53
SAHA-3L	TC	20.60	17.96	0.56
	ST	0	0	0.52
	CT	15.45	10.26	0.59
	SC	20.60	10.26	0.58
	CS	20.60	12.83	0.58
MS-275-4L	TC	10.30	7.70	0.52
	ST	10.30	5.13	0.49
	CT	5.15	2.57	0.53
	SC	10.30	5.13	0.48
	CS	15.45	7.70	0.48

* TC, TanimotoCombo; ST, ShapeTanimoto; CT, ColorTanimoto; SC, ScaledColor; and CS, ComboScore.

**Table 6:** Ligand Enrichments of four pipelines in terms of ROCE 0.5%, ROCE 1%, and ROC AUC.

Pipeline	ROCE 0.5%	ROCE 1%	ROC AUC
Hypo1_FRED_SAHA-3	72.10	38.49	0.87
Hypo1_FRED_Apo	56.65	33.36	0.87
Hypo1_GOLD_Apo	0	11.20	0.65
Hypo1_LibDock_Apo	5.15	7.70	0.86

## Abbreviations

HDAC3	histone deacetylase 3
VS	virtual screening
SBVS	structure-based VS
LBVS	ligand-based VS
HDACs	histone deacetylases
HATs	histone acetyltransfereases
NF-κB	nuclear factor κB
HDAC3Is	HDAC3 inhibitors
CADD	computer-aided drug design
MUBD-HDACs	maximal unbiased benchmarking datasets for histone deacetylases
DS	Discovery Studio
R	aromatic ring
H	hydrophobic group
D	H-bond donor
A	H-bond acceptor
ZBG	zinc binding group
TC	TanimotoCombo
ST	ShapeTanimoto
CT	ColorTanimoto
SC	ScaledColor
CS	ComboScore
ROC	receiver operating characteristic
AUC	area under the curve
ROCE	ROC enrichment
